# Regional wall function before and after acute myocardial infarction; an experimental study in pigs

**DOI:** 10.1186/1471-2261-14-118

**Published:** 2014-09-13

**Authors:** Ulrika S Pahlm, Joey FA Ubachs, Einar Heiberg, Henrik Engblom, David Erlinge, Matthias Götberg, Håkan Arheden

**Affiliations:** Department of Clinical Physiology, Clinical Sciences, Lund University Hospital, SE-22185 Lund, Sweden; Department of Cardiology, Catharina Hospital Eindhoven, Eindhoven, The Netherlands; Centre for Mathematical Sciences, Lund University, Lund, Sweden; Department of Biomedical Engineering, Faculty of Engineering, Lund University, Lund, Sweden; Department of Cardiology, Clinical Sciences, Lund University, Lund, Sweden

## Abstract

**Background:**

Left ventricular function is altered during and after AMI. Regional function can be determined by cardiac magnetic resonance (CMR) wall thickening, and velocity encoded (VE) strain analysis. The aims of this study were to investigate how regional myocardial wall function, assessed by CMR VE-strain and regional wall thickening, changes after acute myocardial infarction, and to determine if we could differentiate between ischemic, adjacent and remote segments of the left ventricle.

**Methods:**

Ten pigs underwent baseline CMR study for assessment of wall thickening and VE-strain. Ischemia was then induced for 40-minutes by intracoronary balloon inflation in the left anterior descending coronary artery. During occlusion, ^99m^Tc tetrofosmin was administered intravenously and myocardial perfusion SPECT (MPS) was performed for determination of the ischemic area, followed by a second CMR study. Based on ischemia seen on MPS, the 17 AHA segments of the left ventricle was divided into 3 different categories (ischemic, adjacent and remote). Regional wall function measured by wall thickening and VE-strain analysis was determined before and after ischemia.

**Results:**

Mean wall thickening decreased significantly in the ischemic (from 2.7 mm to 0.65 mm, p < 0.001) and adjacent segments (from 2.4 to 1.5 mm p < 0.001). In remote segments, wall thickening increased significantly (from 2.4 mm to 2.8 mm, p < 0.01). In ischemic and adjacent segments, both radial and longitudinal strain was significantly decreased after ischemia (p < 0.001). In remote segments there was a significant increase in radial strain (p = 0.002) while there was no difference in longitudinal strain (p = 0.69). ROC analysis was performed to determine thresholds distinguishing between the different regions. Sensitivity for determining ischemic segments ranged from 70-80%, and specificity from 72%-77%. There was a 9% increase in left ventricular mass after ischemia.

**Conclusion:**

Differentiation thresholds for wall thickening and VE-strain could be established to distinguish between ischemic, adjacent and remote segments but will, have limited applicability due to low sensitivity and specificity. There is a slight increase in radial strain in remote segments after ischemia. Edema was present mainly in the ischemic region but also in the combined adjacent and remote segments.

## Background

Acute myocardial infarction (AMI) is a major cause of death worldwide despite diagnostic and therapeutic improvements [1]. Mortality is especially high in patients with AMI and out of hospital cardiac arrest.

Regional left ventricular function is altered during and after AMI. This includes changes in the infarcted and ischemic regions as well as stunning in adjacent and remote areas of the myocardium [2–6]. Most studies describe changes in the infarcted myocardium while there is less information about changes in remote myocardium. It is still somewhat controversial whether remote myocardium after AMI is hypo-functioning [6] or hyper-functioning [7]. This has not been well studied in the hyper acute setting.

Cardiac magnetic resonance (CMR) is a comprehensive diagnostic tool that can provide accurate and reproducible measurements of cardiac volumes [8], dimensions [8], regional cardiac function [9, 10] and infarct size [11, 12]. It has emerged as the gold standard for assessing systolic wall thickening [10]. Studies have shown that regional wall function can be assessed using CMR strain analysis [13, 14]. Strain is a measure of the change in size and shape of an object and can be derived from CMR by using grid-tagging [15], displacement encoding with stimulated echoes (DENSE) [16] or velocity-encoded (VE) imaging [14, 17].

Myocardial function in patients with AMI reaching the hospital has been well studied [5, 6, 18]. Without knowledge of the pre-AMI function it precludes a detailed quantitative analysis of absolute and relative changes in function. The function in the superacute stage (hours) of infarction and in those suffering out of hospital cardiac death is also unknown.

Therefore, the aim of this study was to investigate how regional myocardial wall function, assessed by CMR velocity encoded strain and regional wall thickening, changes after acute myocardial infarction. In order to quantify absolute and relative regional changes we used an experimental pig model with induced ischemia and reperfusion using each animal as its own control. We also aimed to find out if we could differentiate between ischemic, adjacent and remote myocardium as determined by myocardial perfusion MPS by looking at regional myocardial function.

## Methods

### Animal preparation

The study conforms to the Guide for the Care and Use of Laboratory Animals, US National Institute of Health (NIH Publication No. 85–23, revised 1996) and was approved by the Ethics Committee of Lund University, Sweden.

Ten domestic pigs weighing 40–50 kg were fasted overnight with free access to water and all were premedicated with 2 mg/kg azaperone (Stresnil; Leo, Helsingborg, Sweden) administered intramuscularly 30 minutes before the procedure. Induction of anesthesia was performed with 5–25 mg/kg of thiopental (Pentothal; Abbott, Stockholm, Sweden). Administration of the anaesthetic was complemented with intermittent doses of meprobamat (Mebumal; DAK, Copenhagen, Denmark) and thiopental, if needed. Prior to inducing ischemia all pigs underwent a baseline CMR for assessment of wall thickening and velocity encoded strain. Ischemia was induced with inflation of an angioplasty balloon in the left anterior descending coronary artery distal to the first diagonal branch for 40 minutes. An angiogram was performed after inflation of the balloon and before deflation of the balloon in order to verify total occlusion of the coronary vessel and correct balloon positioning. After deflation of the balloon, a second angiogram was performed to verify restoration of blood flow in the previously occluded artery. During occlusion of the artery, ^99m^Tc tetrofosmin was administered intravenously prior to reperfusion and MPS was performed 2–3 hours after occlusion for determination of the area subjected to ischemia. A second CMR examination was performed approximately 3–4 hours after reperfusion. After the second CMR examination the animals were euthanized.

### CMR imaging and analysis

Magnetic resonance imaging was performed on a Philips Intera CV 1.5 T (Philips, Best, the Netherlands) with a five element cardiac synergy coil before and after ischemia. All pigs were placed in supine position and scout images in the three orthogonal planes were acquired as guidance for determination of the standard imaging planes.

### Wall thickening

For assessment of regional wall thickening steady state free precession (SSFP) cine images were acquired in the short-axis plane covering the entire left ventricle from base to apex. Images were also acquired in the 2, 3 and 4 chamber imaging planes. Image parameters were: repetition time 3.2 ms, echo time 1.6 ms, flip angle 60°, image resolution 1.36 × 1.36, slice thickness 8 mm, retrospective ECG gated reconstruction.

From the short-axis cine images systolic wall thickness, wall thickening, and fractional wall thickening (defined as wall thickening divided by end diastolic wall thickness) were assessed before and after ischemia by manual tracing of the endocardial and epicardial borders. The left ventricle was divided in the American Heart Association 17 segment model. Papillary muscles were excluded from the myocardium. The most basal slice included in the analysis was the most basal short-axis slice containing myocardium in 360° of the left ventricular myocardial circumference in end-systole.

### Myocardial strain

All 2D in-plane velocity encoded data was acquired in the 2, 3 and 4 chamber imaging planes. Imaging parameters were: repetition time 23.4 ms echo time 4.6 ms, velocity encoding gradient 20 cm/s, flip angle 15°. Image resolution was typically 1.6 × 1.6 mm, and slice thickness 7 mm with 18–22 time frames per cardiac cycle, retrospective ECG gating.

**Figure 1:**
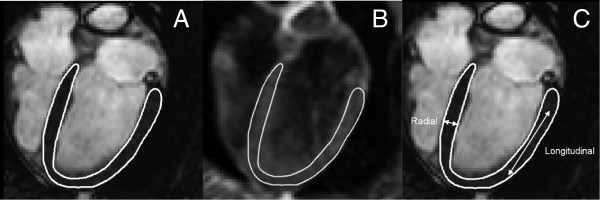
**Outlining the left ventricle. A**. Cine 4 chamber view of the heart before occlusion of LAD. The left ventricle is delineated in white in end-diastole. **B**. Velocity-encoded strain image of the heart in end-diastole, where the white line represents the left ventricle delineation as exported from the cine 4 chamber view. **C**. Illustration of radial and longitudinal strain directions.

We used a previously validated method for VE strain analysis [14]. In short, the myocardium was manually segmented in the 2, 3 and 4 chamber SSFP cine images in end-diastole. Thereafter, the segmentation was exported to the 2D in-plane velocity encoded images and endocardial and epicardial borders were tracked in each time frame throughout the cardiac cycle using the acquired velocity information using an optimization scheme (Figure 1). In one dimension, strain is defined as the fractional change in length of an object. In two dimensions, strain is represented as a 2-dimensional tensor. As the myocardium deforms during the cardiac cycle, a particular myocardial region may be lengthening in the radial direction, while shortening in the circumferential or longitudinal directions. The radial and longitudinal strain directions are depicted in Figure 1 panel C. In the current study, the 2D in-plane velocity data was used to obtain longitudinal and radial strain for assessment of regional myocardial function on a per pixel basis. Strain for each pixel in the myocardium was then colour coded and transformed into colour coded polar plots using the AHA 17 segment model [19].

### Late gadolinium enhancement (LGE)

An extracellular contrast agent (gadopentetate dimeglumine, Bayer Pharma, Berlin, Germany) was administered intravenously at 0.2 mmol/kg 15 minutes before late gadolinium enhancement (LGE) images were acquired. Standard clinical imaging parameters were used for LGE imaging covering the left ventricle from base to apex by using an inversion-recovery gradient-echo sequence (slice thickness, 8 mm; field of view, 340 mm; repetition time, 3.14 ms; echo time, 1.58 ms), with manually adjustment of the inversion time to null the signal from viable myocardium.

The area of hyperenhancement was defined on the LGE short-axis images and was quantified using a previously described and validated semi-automatic algorithm [20] incorporating manual adjustments. Finally, all LGE data was transformed into polar plots according to a 17 segment model [21].

### MPS imaging and analysis

Five hundred MBq of ^99m^Tc tetrofosmin (Amersham Health, Buckinghamshire, UK) was administered intravenously ten minutes before deflation of the angioplasty balloon. The pigs were then imaged in a supine position using a dual head camera (ADAC Vertex, Milpitas, CA, USA) at 32 projections (40s per projection) with a 64 x 64 matrix yielding a digital resolution of 5 x 5 x 5 mm. Short- and long-axis images, covering the left ventricle, gated to ECG, were then reconstructed.

For MPS analysis, automatic segmentation of the LV was performed [22]. In short, the automatic segmentation finds the centerline through the LV wall and identifies the endocardium and epicardium based on an individually estimated wall thickness and signal intensity values within the image. Following delineation, the ischemic area was assessed in contiguous short-axis slices from base to apex using a method for semi-automatic quantification [23]. All myocardium below a threshold of 50 percent of the maximum counts was considered ischemic and expressed as a percentage of the LV volume. Manual adjustment of the automatic delineation was sometimes required in the LV outflow region. Finally, the MPS delineations with ischemia were transformed into colour coded blacked-out polar plots.

All image analyses were performed using an in-house developed freely available software (Segment v1.8 R2860; http://segment.heiberg.se) [24].

### Definition of left ventricular areas: ischemic, adjacent and remote myocardium

**Figure 2:**
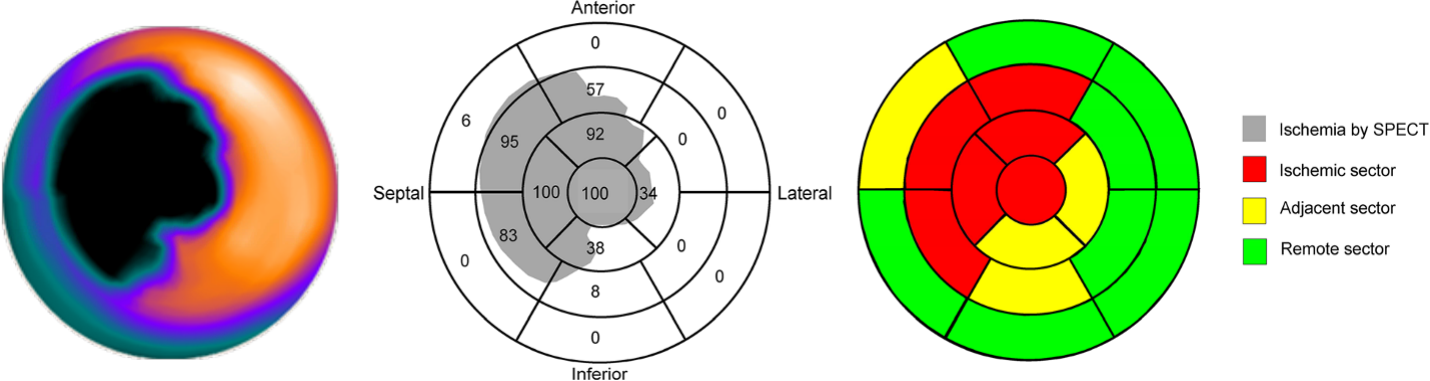
**Left panel shows a MPS polar plot displaying region of ischemia in black.** Middle panel shows AHA 17 segment model of the left ventricle with ischemic region indicated in gray and% of ischemia. Right panel shows colour coded final classification.

In this study, the left ventricle was divided in the AHA 17-segment model for analysis [19]. The 17 segments of the myocardium were divided into 3 groups according to the amount of ischemia seen on MPS. 1) Segments where >50% of the myocardium was ischemic where considered ischemic segments, 2) segments where 1–50% of the myocardium was ischemic were considered adjacent segments and 3) segments with no ischemia were considered remote. The 50% threshold was determined after a consensus discussion. If a segment contained more ischemic than non-ischemic myocardium it was considered ischemic and otherwise it was considered adjacent. Sectors that contained no ischemia were considered remote. The process is illustrated in Figure 2. Myocardial salvage index (MSI) was defined as MSI = 1-(infarct size by LGE)/(ischemic volume by MPS) in a similar fashion as described earlier [25].

### Statistical methods

Wilcoxon Sign Rank test was used to assess changes in volumes, ejection fraction, cardiac output and heart rate. For changes in regional CMR wall thickening and VE strain we used a paired t-test. For all analysis, a p-value below 0.05 was considered significant. Values are expressed as mean ± SEM. To find thresholds to be able to discriminate between ischemic, adjacent and remote areas ROC analysis was performed. The ROC analysis was performed by evaluating sensitivity and specificity for different thresholds of wall thickening, radial strain, and longitudinal strain, respectively. The thresholds was tested in 1000 steps from minimum value to maximum value for each parameter.

## Results

Table 1 shows the CMR measurements for all 10 pigs before and after induced ischemia. After ischemia, there was a significant decrease in stroke volume and ejection fraction associated with a significant increase in heart rate, preserving the cardiac output. The mean myocardial salvage index was 25 ± 15%.

**Table 1:** **Heart rate, cardiac volumes, ejection fraction (EF) and cardiac output (CO) before and after ischemia**

	Before ischemia	After ischemia	p-value
Heart rate [bpm]	81 ± 21	113 ± 30	0.03
Left ventricular mass [ml]	86 ± 7	94 ± 15	0.01
End diastolic volume [ml]	82 ± 11	71 ± 10	0.04
End systolic volume [ml]	47 ± 7	48 ± 9	0.50
Stroke volume [ml]	36 ± 8	23 ± 5	0.01
Ejection fraction [%]	44 ± 7	32 ± 6	0.01
Cardiac output [l/min]	2.8 ± 0.7	2.6 ± 0.8	0.56

There was a significant increase in left ventricular mass from 86 ± 7 ml to 94 ± 15, an increase in 9% (p = 0.01) after ischemia. The increase in mass was not homogenous, and the increase in ischemic areas was 38% ± 3% (mean ± SEM) (p < 0.01), adjacent was 4% ± 2% (mean ± SEM) (p = ns), and in remote areas the increase was 7% ± 3% (mean ± SEM) (p = ns). When combining the results from remote and adjacent areas there was an increase in mass of 6% ±2% (p = ns).

There was no statistically significant difference in wall thickening between the ischemic, adjacent and remote groups before induced ischemia.

**Figure 3:**
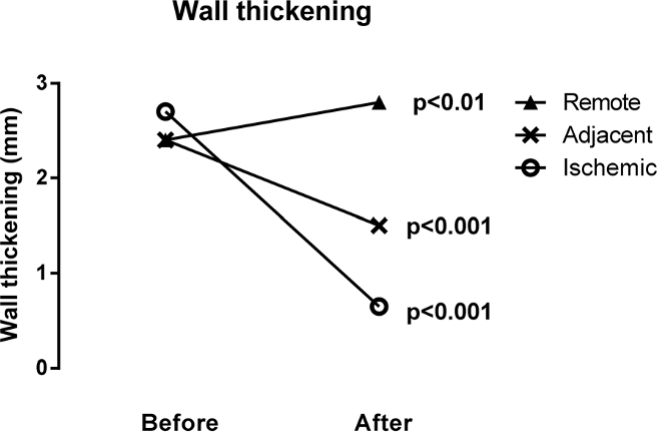
**Wall thickening in mm in ischemic, adjacent and remote areas of the left ventricle before and after ischemia.**

After reperfusion, the mean wall thickening decreased significantly in the ischemic (from 2.7 mm before ischemia to 0.65 mm, p < 0.001) and adjacent segments (from 2.4 to 1.5 mm p < 0.001). In remote myocardium, however, wall thickening increased significantly (from 2.4 mm to 2.8 mm, p < 0.01). Figure 3 shows wall thickening before and after ischemia. Fractional wall thickening was 8% in ischemic, 22% in adjacent, and 36% in remote sectors, respectively. Mean end-diastolic thickness was 10.5 mm in ischemic, 9.4 mm in adjacent, and 8.7 mm in remote sectors, respectively. Mean end-systolic thickness was 11.1 mm in ischemic, 11.4 mm in adjacent, and, 11.8 mm in remote sectors, respectively.

**Figure 4:**
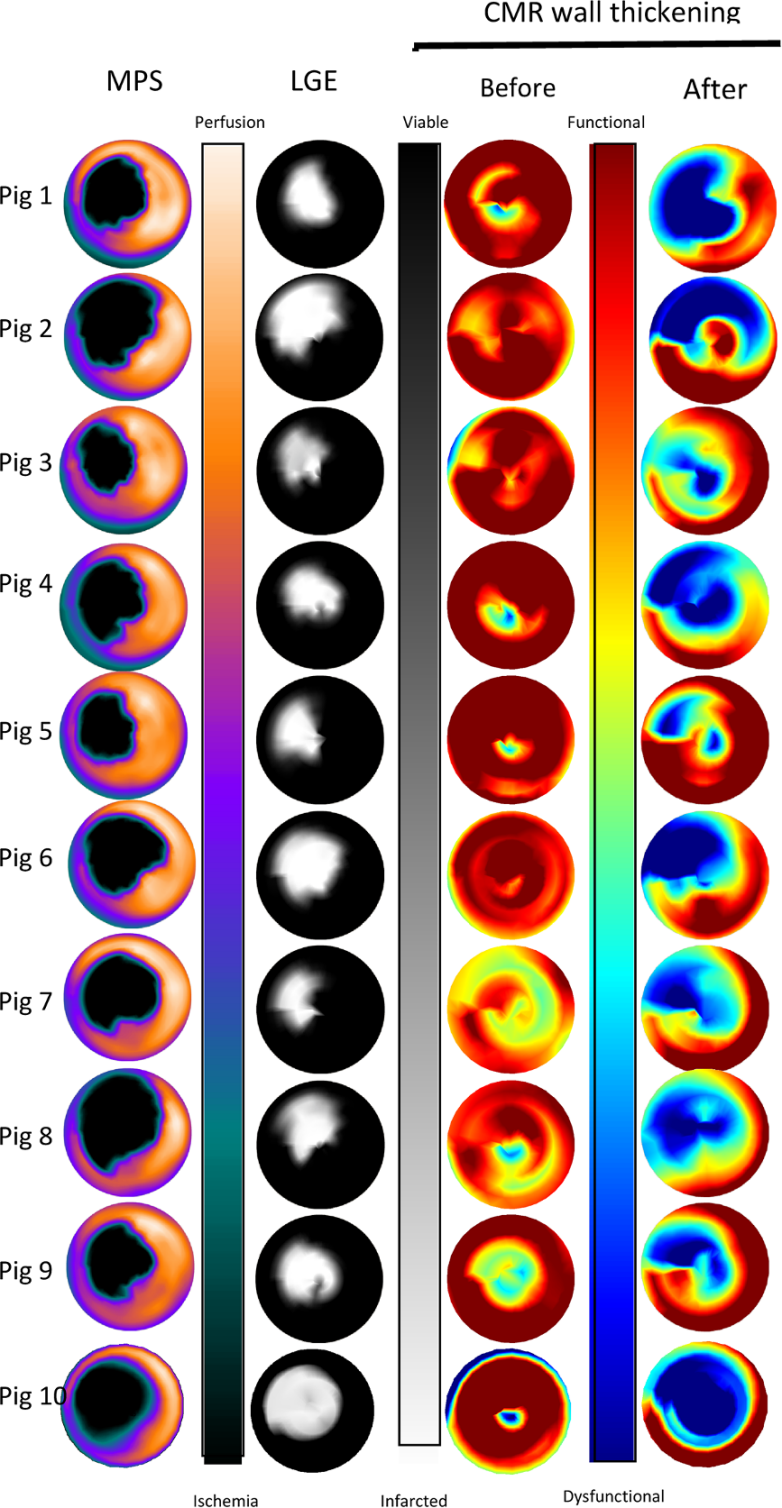
**Polar plots of MPS, LGE and CMR absolute wall thickening for each subject.** The first column shows the ischemic area by MPS. The second column shows infarct transmurality assessed by LGE. The third column shows cardiac function by CMR absolute wall thickening before ischemia and the fourth column cardiac function by CMR absolute wall thickening after ischemia. The color bars next to the respective polar plots denote the scale.

Figure 4 shows polar plots indicating ischemia by MPS, myocardial infarction by LGE, and wall thickening by cine-CMR for each study subject. Dysfunctional myocardium as assessed by CMR absolute wall thickening after ischemia was mainly present in the myocardial region supplied by the left anterior descending artery, similar to MPS and LGE. In the majority of the pigs, however dysfunctional myocardium extended to the adjacent and remote regions of the myocardium.

Before ischemia there was no statistically significant difference in radial or longitudinal strain in the later ischemic, adjacent and remote regions (p > 0.05).

**Figure 5:**
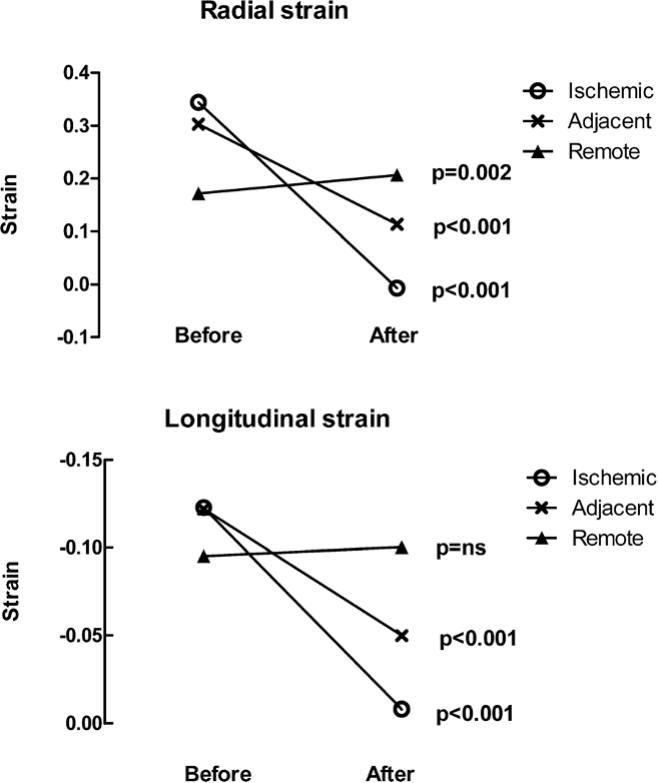
**The upper panel shows radial strain in ischemic, adjacent and remote areas before and after ischemia.** The lower panel shows longitudinal strain before and after ischemia.

Figure 5 shows radial and longitudinal strain before and after ischemia. There was a strong correlation between global wall thickening and global radial strain (r = 0.86, p < 0.001). In ischemic and adjacent areas, both radial and longitudinal strain was significantly decreased after ischemia (p < 0.001). In remote myocardium, there was a significant increase in radial strain (p = 0.002), while there was no difference in longitudinal strain (p = 0.69).

There was a significant decrease in both radial and longitudinal strain between the ischemic and remote myocardium (both p < 0.0001) and between the ischemic and adjacent myocardium (radial p < 0.001, longitudinal p = 0.03) between adjacent and remote myocardium (radial p < 0.0001, longitudinal p < 0.001).). The regional function expressed as radial strain in sectors defined as salvaged (i.e. containing any ischemic myocardium but no infarct) was 12 ± 10%. There was no significant difference between regional strain in salvaged sectors versus adjacent sectors (p = 0.79).

**Figure 6:**
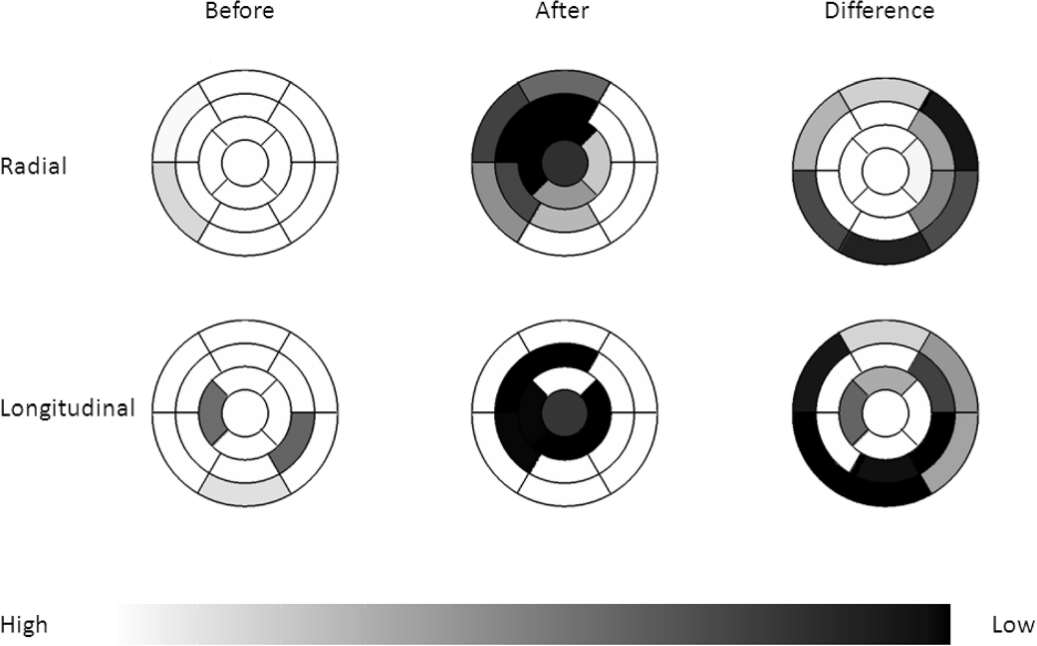
**Mean regional function for all subjects expressed as radial strain (top row) and longitudinal strain (bottom row).** Left column shows high regional function before ischemia. Middle column shows decreased regional function in segments supplied by left anterior descending artery. Right column shows difference in regional function before and after ischemia. White means high strain and black low strain.

Figure 6 shows that regional function measured by radial and longitudinal strain decreases most significantly in left ventricular areas supplied by the left anterior descending artery.

**Figure 7:**
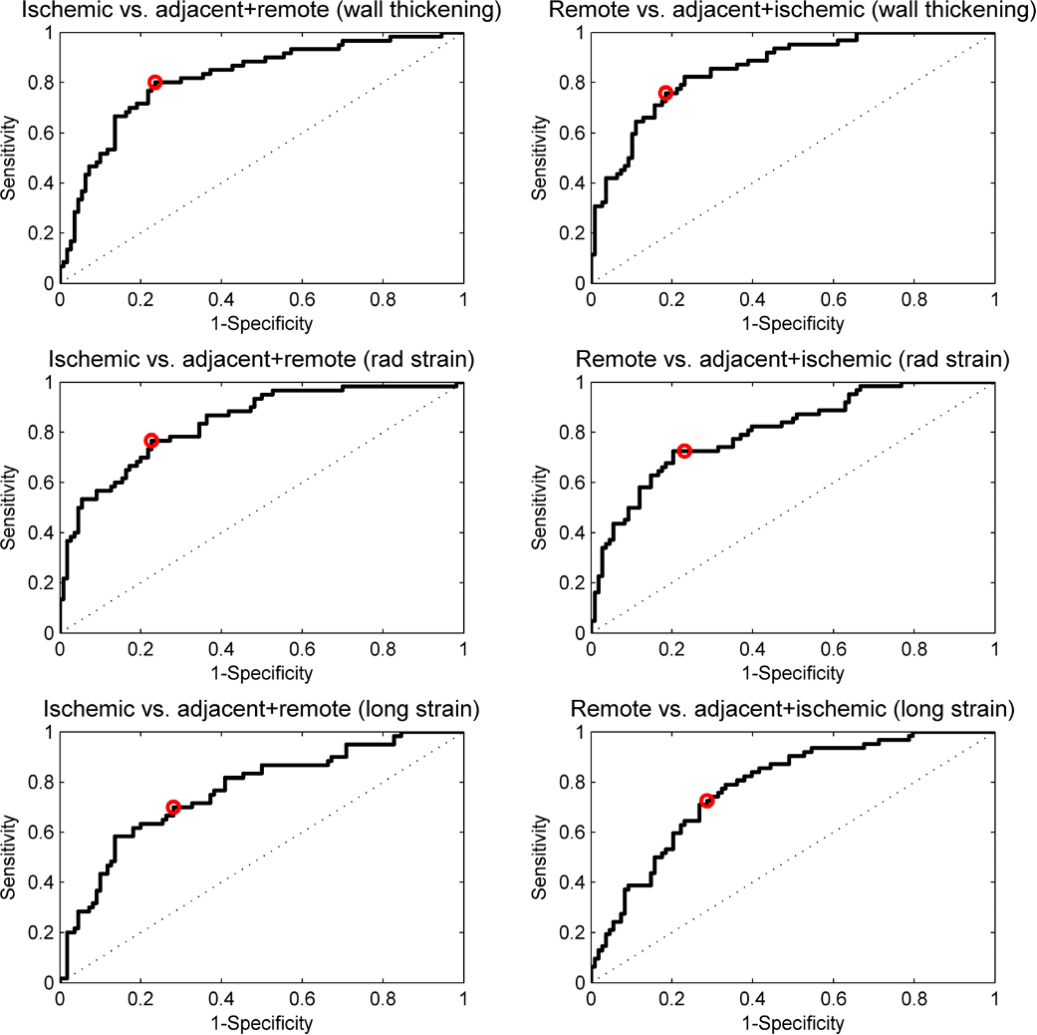
**ROC analysis determining threshold.** Upper panel shows results for wall thickening. Middle panel shows results for radial strain. Lower panel shows results for longitudinal stain. Left column shows differentiation between ischemic and non-ischemic areas. Right column shows differentiation between remote and non-remote areas. Red marker shows sensitivity and specificity for optimal thresholds.

Figure 7 shows ROC curves for thresholds. Ischemic sectors were compared to adjacent and remote sectors combined, and remote sectors were compared to ischemic and adjacent sectors combined. The results of the ROC analysis are presented in Table 2.

**Table 2:** **Thresholds for wall thickening, radial strain and longitudinal strain determined by ROC analysis for differentiations between ischemic, adjacent and remote areas**

	Threshold	Sensitivity%	Specificity%
Wall thickening ischemic	< 1.4 mm	80	76
Wall thickening adjacent	1.4 - 2.1 mm	35	90
Wall thickening remote	>2.1 mm	76	81
Radial strain ischemic	< 0.06	77	77
Radial strain adjacent	0.06-0.15	33	89
Radial strain remote	>0.15	73	77
Long. strain ischemic	> −0.04	70	72
Long. strain adjacent	−0.04 - -0.07	15	88
Long. strain remote	<−0.07	73	71

## Discussion

In this study we found that there is a significant decrease in regional wall function in the ischemic and adjacent segments of the left ventricle measured by CMR wall thickening and VE-strain after induced myocardial infarction. There is a slight increase in regional wall function seen by wall thickening and VE radial strain in remote myocardium. The non-significant increase in longitudinal strain seen in the remote myocardium was likely attributed to the limited number of subjects.

The decreased function in the ischemic region is well known and has obvious causes.

The reduced regional function in adjacent areas have been described previously [2] and represents myocardial stunning as described by Braunwald et al. [3, 4]. This prolonged dysfunction remain present for hours, days or even a few weeks after ischemia and may be explained by the presence of edema [26]. Similar results were demonstrated by Engblom et al. in a human population [27]. The significant decrease in EDV in conjunction with unaffected ESV after ischemia suggests diastolic dysfunction as a result of the ischemia. The decreased regional function in ischemic regions indicates regional systolic dysfunction.

In this study we found that there was a slight, but statistically significant, increase in regional myocardial function in remote areas measured both by wall thickness and radial strain. There was, however, no change in function seen by longitudinal strain. Increased function in remote areas has been described previously [6, 7, 28] but most other studies have found a decreased function in both animals [29, 30] and humans [6, 27, 31, 32]. Reasons for the lack of consensus between studies are unknown but may be related to duration of ischemia, reperfusion and timing of imaging. An advantage of our study is the analysis of strain and wall thickening measurements in the same animal both prior to and after infarction. The presented method for measuring regional left ventricular function in ischemic, adjacent and remote areas may be used in controlled experimental settings to investigate the effect of cardioprotective treatments, such as cooling [33].

In a direct comparison between strain tagging and wall thickening, a previous study by Götte et al. [34] using CMR has shown that the former was more accurate in discriminating infarcted from remote myocardium. In our study, however, we found that wall thickening and radial VE strain were similar in discriminating between ischemic and non-ischemic areas. We found that both wall thickening and strain are able to differentiate between ischemic and non-ischemic, remote and non-remote myocardium, respectively. However, the sensitivity to detect adjacent regions was poor, 33% for wall thickening and 15% for strain. The thresholds have limited applicability due to the low sensitivity and specificity. This has implications on trying to differentiate between remote, adjacent and ischemic regions based on regional function regardless of modality. The detection of adjacent sectors alone may be of limited clinical value, however the rational for including adjacent sectors in this study was to differentiate between sectors with high grade ischemia (ischemic sectors) and low grade ischemia (adjacent sectors).

We also found an increase of left ventricular mass of 9% following ischemia that is likely caused by edema. The increase was impressive in the ischemic area, measuring 37% ± 3% (p < 0.01) compared to 6% ± 2 (p = ns) in the combined remote and adjacent areas. This was also supported by end diastolic thickness that was significantly higher in ischemic compared to remote sectors (p < 0.01).

### Limitations

The study was conducted on 10 pigs, all with occlusion of the LAD. How results for CMR wall thickening and VE-strain would be affected by right coronary artery or left circumflex occlusion remains to be studied. In wall thickening, the most basal slices in end-diastole are often excluded since myocardium cannot be found in 360° of the left ventricular myocardial circumference in end-systole due to long-axis AV-plane motion [35]. No standard definition of ischemic, adjacent and remote areas of the ventricle have been established, therefore caution should be taken when comparing results with other studies.

## Conclusions

Thresholds for wall thickening and strain could be established for differentiation between ischemic, adjacent and remote areas. These thresholds, however will have limited clinical applicability due to the low sensitivity and specificity. Regional left ventricular function is reduced in the ischemic and areas adjacent to the ischemia after reperfused anterior myocardial infarction, while there is a slight increase in radial function in remote areas of the left ventricle. Edema was present mainly in the ischemic region but also to a slighter degree in the combined adjacent and remote areas.
